# MAGI2-AS3/miR-450b-5p/COLEC10 interaction network: A potential therapeutic and prognostic marker in hepatocellular carcinoma

**DOI:** 10.1016/j.iliver.2025.100146

**Published:** 2025-02-13

**Authors:** Lan-Qing Yao, Yong-Kang Diao, Jin-Bo Gong, Li-Hui Gu, Jia-Hao Xu, Ming-Da Wang, Chao Li

**Affiliations:** a Department of Hepatobiliary Surgery, Eastern Hepatobiliary Surgery Hospital, Naval Medical University, Shanghai 200438, China; b Department of Hepatobiliary Surgery, Affiliated Hospital of Nantong University, Nantong 226001, Jiangsu, China

**Keywords:** Competitive endogenous RNA, Hepatocellular carcinoma, Bioinformatic analysis, Differentially expressed genes

## Abstract

***Background and aims*:**

Hepatocellular carcinoma (HCC) is a prevalent malignancy with poor prognosis. This study uses integrated bioinformatic analyses to explore potential competing endogenous RNA (ceRNA) network chains in HCC.

***Methods*:**

HCC expression profile data were obtained from the Gene Expression Omnibus dataset, and differential expression analysis was conducted to identify differentially expressed mRNAs (DEmRNAs), microRNAs (DEmiRNAs), and long non-coding RNAs (DElncRNAs) between HCC and normal liver tissue samples. Univariate Cox regression analysis was performed to identify mRNAs associated with the prognosis of HCC patients. Gene Ontology and Kyoto Encyclopedia of Genes and Genomes enrichment analyses were used to classify the identified genes functionally. Cytoscape software was used to construct a protein–protein interaction network. Using the intersection method, a ceRNA network was established to align data from two databases (miRTarBase and miRcode). Pearson correlation analysis was conducted to evaluate the relationships between lncRNAs and mRNAs.

***Results*:**

A total of 106 prognosis-related DEmRNAs were identified between HCC and normal samples. A total of 132 DEmiRNAs and 42 DElncRNAs were dysregulated in HCC. A ceRNA network of three lncRNAs, six miRNAs, and eight mRNAs was constructed. High expression of MCM10, CDKN3, RRM2, KIF3A, and ALYREF correlated with a poor prognosis, while high expression of CPEB2, COLEC10, and PBLD was associated with a better prognosis for HCC patients. Expression analysis confirmed the differential expression of these genes in HCC samples. Correlation analysis revealed that a MAGI2-AS3/hsa-miR-450b-5p/COLEC10 axis might play a crucial role in the progression of HCC.

***Conclusion*:**

The ceRNA network constructed could provide insight into HCC tumorigenesis and might lead to new molecular biomarkers for diagnosing and treating HCC.

## 1 Introduction

Liver cancer is a potentially fatal disease that has a high incidence rate and poor prognosis, with approximately 865,269 new cases diagnosed each year.^1^ The incidence and mortality rates of liver cancer rank sixth and third among tumors globally, respectively, with hepatocellular carcinoma (HCC) accounting for approximately 75%–85% of these cases.^1^ As a common malignant tumor, HCC has been linked to various conditions, including viral infections and liver cirrhosis.^2^ Current treatment options for HCC include surgical resection, liver transplantation, ablation, transarterial chemoembolization, and systemic therapy.^3^ The 5-year overall survival rate for HCC patients who undergo curative treatment can reach 60%–70%.^4^, ^5^, ^6^ However, since liver cancer is predominantly diagnosed at intermediate and advanced stages, only a limited number of patients are eligible for radical treatment.^7^^,^^8^ Consequently, most HCC patients receive only local treatment, resulting in a low 5-year survival rate.^9^^,^^10^ Thus, studies of the molecular mechanisms underlying HCC tumorigenesis and new therapeutic strategies are urgently needed.

Competing endogenous RNAs (ceRNAs) containing miRNA response elements (MREs) play a significant role in cancer progression.^11^^,^^12^ These ceRNAs can compete with miRNAs through their MREs, facilitating interactions between non-coding and coding RNAs within complex ceRNA networks.^13^ Such molecular interactions can potentially influence a broad range of biological processes. Abnormal regulation of the lncRNA-miRNA-mRNA network may promote the occurrence and development of various tumors, such as lung cancer,^14^ colorectal cancer,^15^ gastric cancer,^16^ and glioblastoma.^17^ For example, HOXA-AS2 can negatively regulate miR-885-5p in glioblastoma to enhance RBBP4 expression, thereby promoting carcinogenesis^17^ Additionally, Mesenchymal Stem Cells (MSC)-induced lncRNA HCP5 can inhibit miR-3619-5p and upregulate PPARG coactivator 1α (PPARGC1A), which enhances the transcription complex of peroxisome proliferator-activated receptor (PPAR) coactivator-1α (PGC1α) and CCAAT/enhancer-binding protein beta (CEBPB).^18^ This transcriptional activation induces carnitine palmitoyltransferase 1 (CPT1), thereby promoting fatty acid oxidation (FAO) in gastric cancer cells, contributing to the maintenance of gastric cancer stemness and resistance to chemotherapy.^18^ In HCC, exosomal LncSNHG16 can be phagocytosed by telocytes and downregulate miRNAs by binding to miR-942-3p in telocytes, which induces the upregulation of the target gene MMP9 and promotes metastasis of HCC.^19^ Therefore, identifying functional ceRNA networks in HCC is important for deepening our understanding of HCC and developing new diagnostic and therapeutic strategies.

In this study, we used bioinformatics tools to identify prognostic-related mRNAs, miRNAs, and lncRNAs in HCC and constructed a ceRNA network. Subsequently, we verified the expression of key miRNAs, lncRNAs, and mRNAs in HCC. These findings may offer valuable insights into the pathogenesis of HCC and contribute to more precise clinical diagnosis and treatment strategies.

## 2 Materials and methods

### 2.1 Data collection

Three expression profile datasets (GSE101728, GSE138178, GSE146719) for HCC were downloaded from the National Center for Biotechnology Information Gene Expression Omnibus (https://www.ncbi.nlm.nih.gov/geo/). The GSE101728 dataset included expression profiling of lncRNA and mRNA from seven HCC and seven normal samples. The GSE138178 dataset contained expression profiling of lncRNA and mRNA from 49 HCC and 49 normal samples. The GSE146719 dataset included expression profiling of mRNA and miRNA from three HCC and three normal samples. Additionally, mRNA expression profile data for 374 HCC and 50 normal samples were collected from The Cancer Genome Atlas (TCGA; https://cancergenome.nih.gov/) database.

### 2.2 Differential expression analysis

In the GSE101728, GSE138178, and GSE146719, the differential expression analysis between HCC and normal groups was performed using the “limma (v3.58.1)” package in R language. The differentially expressed mRNAs (DEmRNAs), DEmiRNAs, and DElncRNAs between the two groups were identified by *p* < 0.05 and |log2 fold change| >1. The R package “volcano” and the R package “ComplexHeatmap” were used to draw volcano plots and heat maps, respectively.

### 2.3 Univariate Cox regression analysis

Univariate Cox regression analysis was used to evaluate the impact of related genes on patient survival risk, and a *p* < 0.05 and Hazard Ratio (HR)≠1 were used to identify survival-related risk genes of HCC patients.

### 2.4 Enrichment analysis

The Gene Ontology (GO), including Biological Process, Molecular Function, and Cellular Component functional enrichment analysis and Kyoto Encyclopedia of Genes and Genome (KEGG) pathways enrichment analysis were performed using “ClusterProfiler (v4.12.0)” in R. The significantly enriched GO terms and KEGG pathways were identified using a *p* < 0.05, and the top 30 GO terms and KEGG pathways were examined.

### 2.5 Protein–protein interaction (PPI) network analysis

The Search Tool for the Retrieval of Interacting Genes (STRING) database was used to obtain DEmRNA-encoded protein and PPI information. Moreover, the PPI pairs with comprehensive scores >400 were downloaded and analyzed.

### 2.6 Survival analysis

The prognostic risk of genes in HCC patients was analyzed using the “survival” package. Survival differences between the two groups were analyzed using the “survfit” function. Patients with HCC were divided into high and low gene expression groups using the best cutoff threshold obtained from “surv_cutpoint.” The results were visualized via a Kaplan–Meier curve. All statistical analyses were performed using R version. A two-tailed *p* < 0.05 was considered statistically significant.

### 2.7 Construction of lncRNA-miRNA-mRNA networks

An initial lncRNA-miRNA-mRNA network was constructed based on the ceRNA hypothesis. The miRTarBase database was utilized to predict interactions between the DEmRNAs and DEmiRNAs. Subsequently, the interactions between DElncRNAs and DEmiRNAs were predicted using the miRcode database. Cytoscape (v3.7.2) was employed to construct the preliminary ceRNA network.

### 2.8 Correlation analysis

The correlation between lncRNA and mRNA in the GSE101728 and GSE138178 datasets, and the correlation between miRNA and mRNA in the GSE146719 data set was calculated using the Pearson correlation coefficient.

## 3 Results

### 3.1 Identification of DEmRNAs between HCC and normal tissue samples

**Fig. 1:**
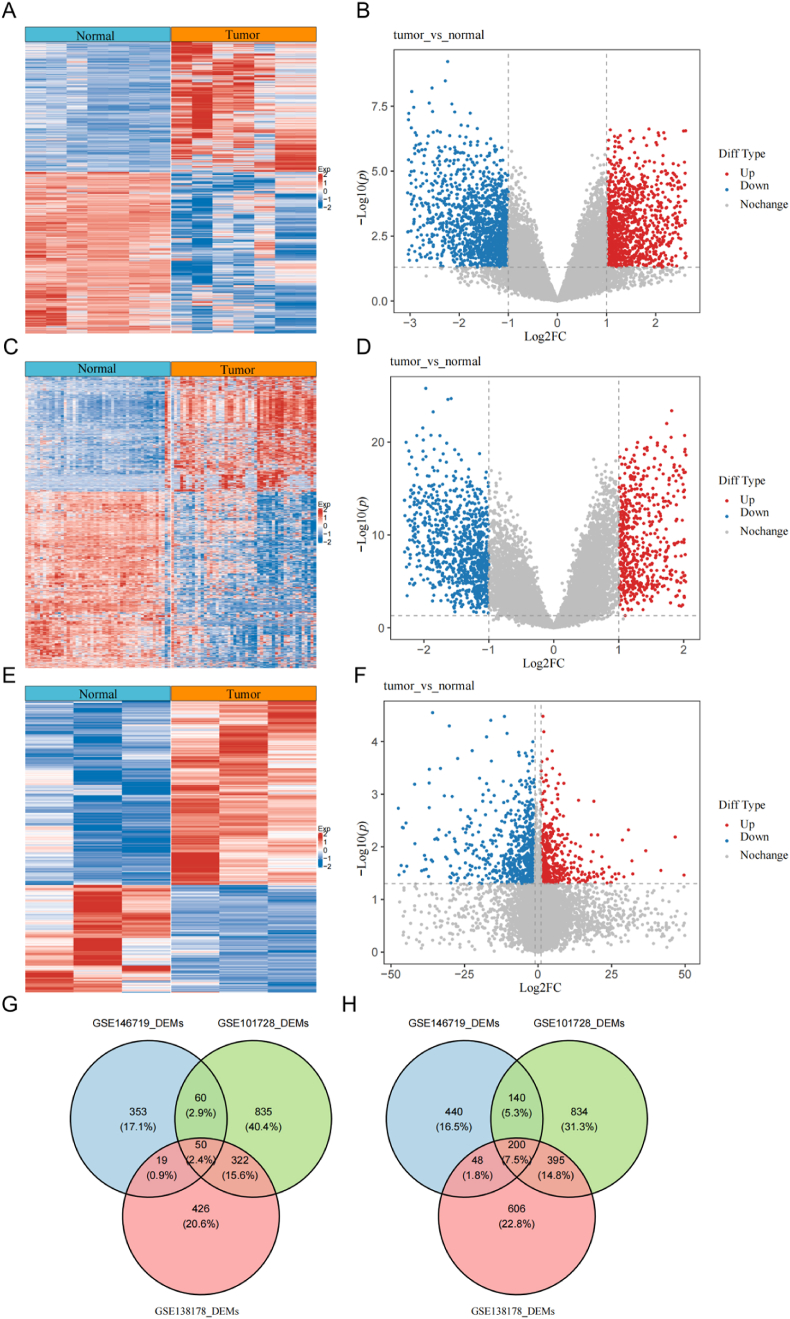
**Identification of differentially expressed genes between hepatocellular carcinoma (HCC) and normal samples**. Heat map (A) and volcano plot (B) of differentially expressed genes between HCC and normal samples in the GSE101728 dataset. Heat plot (C) and volcano plot (D) of differentially expressed genes between HCC and normal samples in the GSE138178 dataset. Heat plot (E) and volcano plot (F) of differentially expressed genes between HCC and normal samples in the GSE146719 dataset. Venn diagram of upregulated differentially expressed genes (G) and downregulated differentially expressed genes (H) across the GSE101728, GSE138178, and GSE146719 datasets.

We first analyzed DEmRNAs in HCC and normal liver samples across the GSE101728, GSE138178, and GSE146719 datasets. In the GSE101728 dataset, 2836 DEmRNAs were identified, comprising 1267 up-regulated genes and 1569 down-regulated genes (HCC vs. normal, *p* < 0.05; Fig. 1A,B). In the GSE138178 dataset, we identified 2066 DEmRNAs, including 817 up-regulated genes and 1249 down-regulated genes (HCC vs. normal, *p* < 0.05; Fig. 1C,D). The GSE146719 dataset revealed 1301 DEmRNAs, with 482 up-regulated genes and 828 down-regulated genes (HCC vs. normal, *p* < 0.05; Fig. 1E,F). Additionally, we identified 50 genes that were up-regulated (Fig. 1G) and 200 genes that were down-regulated (Fig. 1H) in HCC samples compared to normal samples across the GSE101728, GSE138178, and GSE146719 datasets.

### 3.2 Identification of prognosis-related genes in HCC patients

**Fig. 2:**
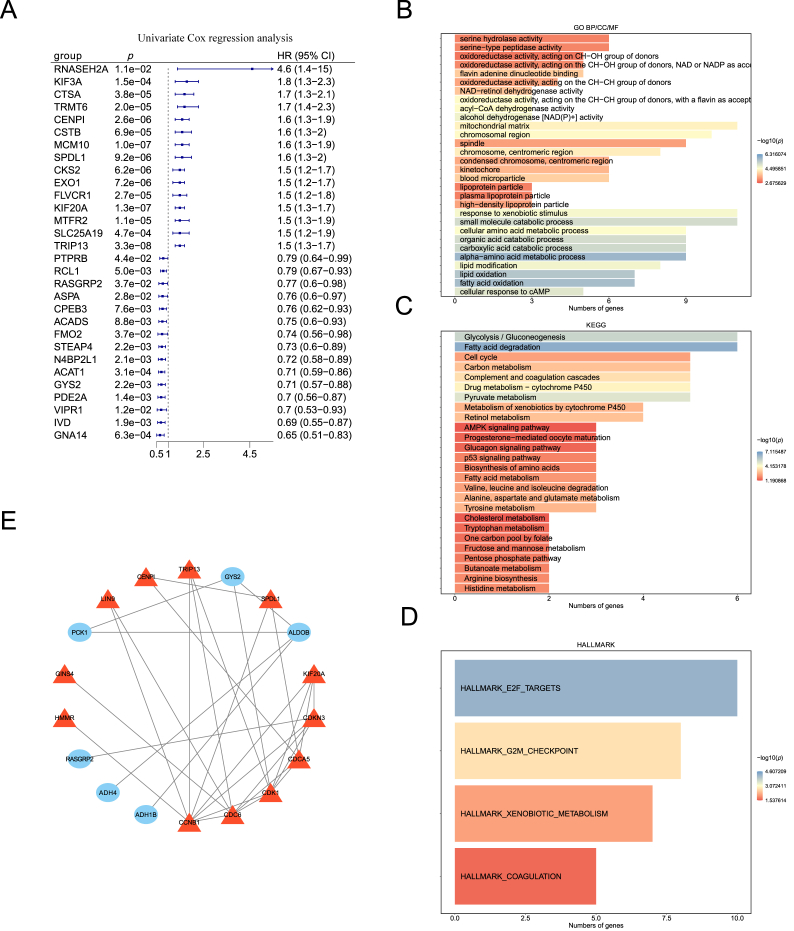
**Univariate Cox regression analysis of prognosis-related genes and functional enrichment analysis.** (A) The univariate Cox regression analysis screened prognosis-related genes. (B) Bar chart of the GO enrichment analysis of the prognosis-related genes. (C) Bar chart of the KEGG pathway enrichment analysis of the prognosis-related genes. (D) Bar chart of the hallmark pathway enrichment analysis of the prognosis-related genes. (E) A PPI network of hub genes was constructed using the STRING online database.

A univariate Cox regression analysis identified 106 prognosis-related genes for HCC among these 250 DEmRNAs between HCC and normal samples (*p* < 0.05, HR≠1; Fig. 2A, Table S1). Furthermore, the GO enrichment analysis revealed that these 106 genes were predominantly enriched in serine hydrolase activity, serine-type peptidase activity, spindle, mitochondrial matrix, and chromosomal region processes (*p* < 0.05; Fig. 2B). The KEGG enrichment analysis indicated that these 106 genes were significantly enriched in glycolysis/gluconeogenesis, fatty acid degradation, cell cycle, carbon metabolism, and complement and coagulation cascade pathways (*p* < 0.05; Fig. 2C). Additionally, the HALLMARK enrichment analysis indicated that these 106 genes were significantly enriched in the E2F targets, G2M checkpoint, xenobiotic metabolism, and coagulation pathways (*p* < 0.05; Fig. 2D).

### 3.3 PPI analysis

To evaluate interactive associations, a total of 106 prognosis-related genes were mapped to the STRING database. Those with an interaction score greater than 400 were entered into Cytoscape for PPI network analysis and visualization. These genes included TRIP13, CDC6, FMO2, PON1, DMGDH, and others (Fig. 2E).

### 3.4 Identification of DEmiRNAs between HCC and normal liver samples

**Fig. 3:**
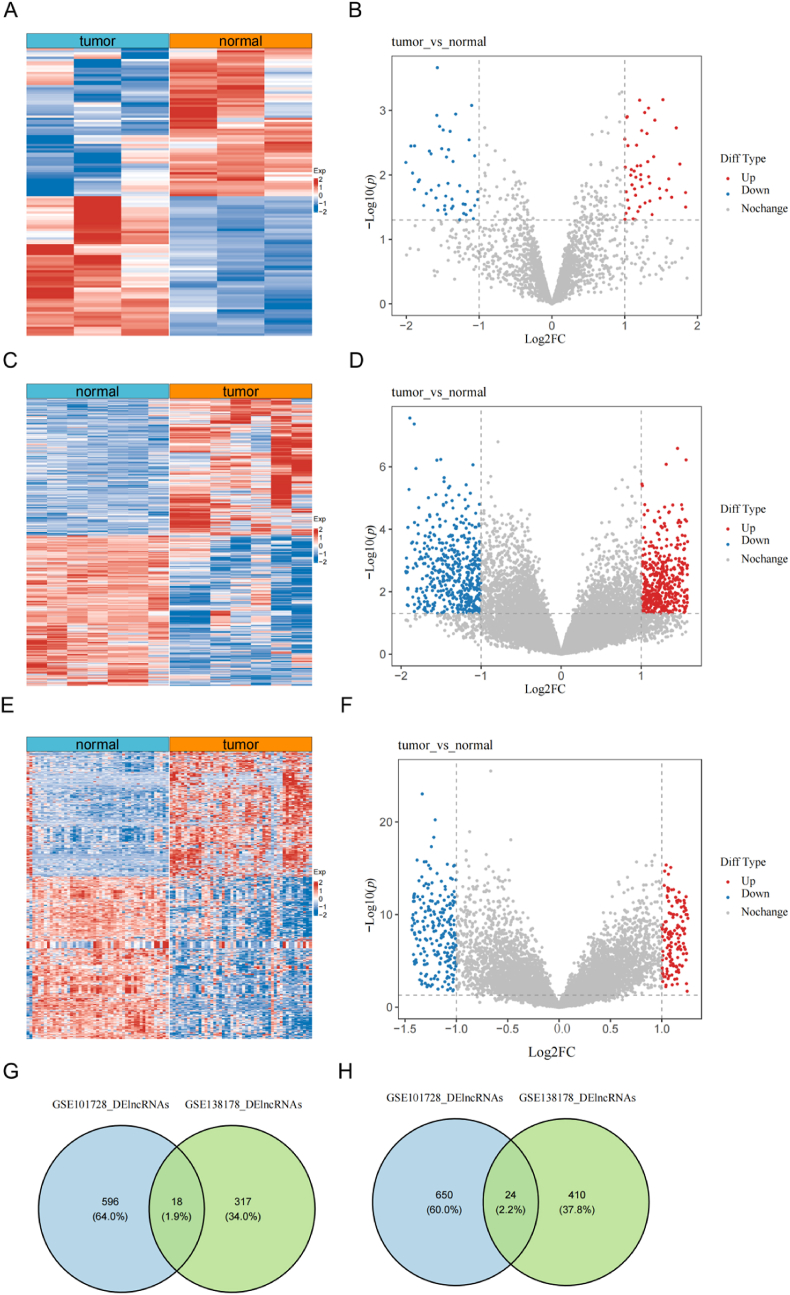
**Identification of differentially expressed miRNAs and lncRNAs between HCC and normal liver samples**. Heat plot (A) and volcano plot (B) of differentially expressed miRNAs between HCC and normal samples in the GSE146719 dataset. Heat plot (C) and volcano plot (D) of differentially expressed lncRNAs between HCC and normal samples in the GSE101728 dataset. Heat plot (E) and volcano plot (F) of differentially expressed lncRNAs between HCC and normal samples in the GSE138178 dataset. Venn diagram of upregulated differentially expressed genes (G) and downregulated differentially expressed lncRNAs (H) across GSE101728 and GSE138178 datasets.

Research has confirmed the molecular mechanisms underlying the ceRNA regulatory network in the occurrence and progression of various cancers.^1^ However, few bioinformatic analyses have constructed ceRNA networks specifically for HCC. To construct a ceRNA network, we first identified the miRNAs differentially expressed between HCC and normal. In the GSE146719 dataset, we analyzed DEmiRNAs between HCC and normal samples and discovered 132 DEmiRNAs, including 64 up-regulated and 68 down-regulated miRNAs (HCC vs. normal, *p* < 0.05; Fig. 3A,B).

### 3.5 Identification of DElncRNAs between HCC and normal liver samples

In the GSE101728 and GSE138178 datasets, we analyzed DElncRNAs between HCC and normal samples. In the GSE101728 dataset, 1288 DElncRNAs were identified, comprising 614 up-regulated lncRNAs and 674 down-regulated lncRNAs (HCC vs. normal, *p* < 0.05; Fig. 3C,D). The GSE138178 dataset revealed 769 DElncRNAs, with 335 up-regulated lncRNAs and 434 down-regulated lncRNAs (HCC vs. normal, *p* < 0.05; Fig. 3E,F). Furthermore, across both datasets, we identified 18 lncRNAs that were up-regulated (Fig. 3G) and 24 lncRNAs that were down-regulated (Fig. 3H) in HCC samples compared to normal samples.

### 3.6 Construction of a ceRNA network

**Fig. 4:**
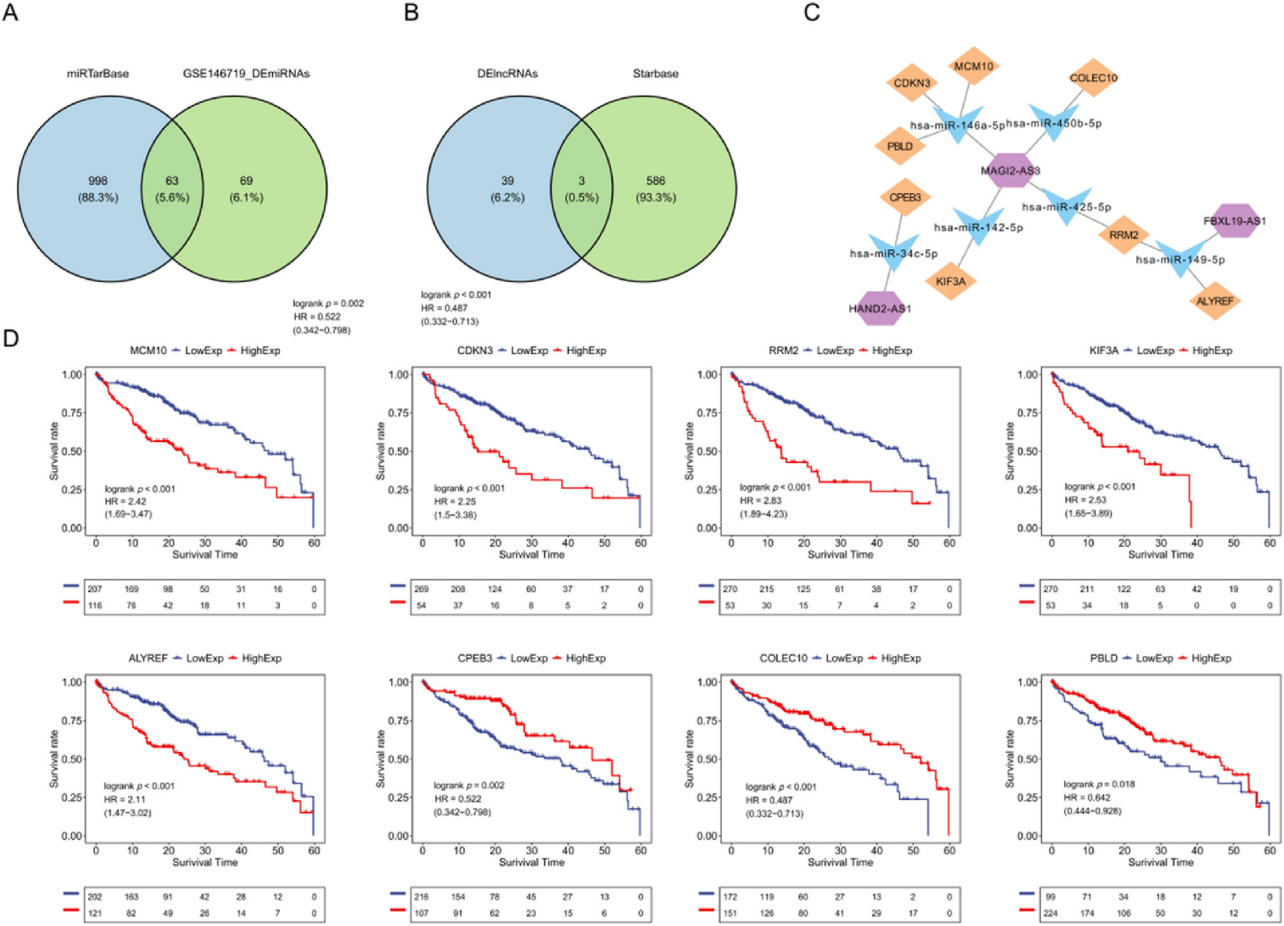
**Construction of a ceRNA network.** (A) Venn diagram showing the intersection of DEmiRNAs and predicted miRNAs. (B) Venn diagram showing the intersection of DElncRNAs and predicted lncRNAs. (C) The lncRNA-miRNA-mRNA ceRNA network. (D) Kaplan–Meier curve of HCC patients with high and low gene groups.

First, we predicted 1061 upstream miRNAs of 106 prognosis-related genes using the miRTarbase database. We found that 63 miRNAs were differentially expressed between HCC and normal samples (Fig. 4A). A total of 589 lncRNAs binding to the 63 miRNAs were predicted in the MiRcode website. Three lncRNAs (BXL19-AS1, HAND2-AS1, and MAGI2-AS3) were differentially expressed between HCC and normal samples (Fig. 4B). Among the 63 DEmiRNAs, six miRNAs—hsa-miR-146a-5p, hsa-miR-450b-5p, hsa-miR-34c-5p, hsa-miR-142-5p, hsa-miR-425-5p, and hsa-miR-149-5p—interacted with three lncRNAs. The target genes of these six miRNAs included MCM10, CDKN3, RRM2, KIF3A, ALYREF, CPEB2, COLEC10, and PBLD. miRNA–lncRNA–mRNA networks were constructed based on the three lncRNAs, six miRNAs, and eight mRNAs (Fig. 4C). Furthermore, in the TCGA cohort, we analyzed the role of these eight mRNAs in the prognosis of HCC patients and found that high expression of MCM10, CDKN3. RRM2, KIF3A, and ALYREF were correlated with poor prognosis of HCC patients, while high expression of CPEB2, COLEC10, and PBLD was associated with better prognosis (*p* < 0.05; Fig. 4D). These results suggest that these eight mRNAs are closely correlated with prognosis of HCC patients.

### 3.7 Expression of mRNAs, miRNAs, and lncRNAs in HCC samples

**Fig. 5:**
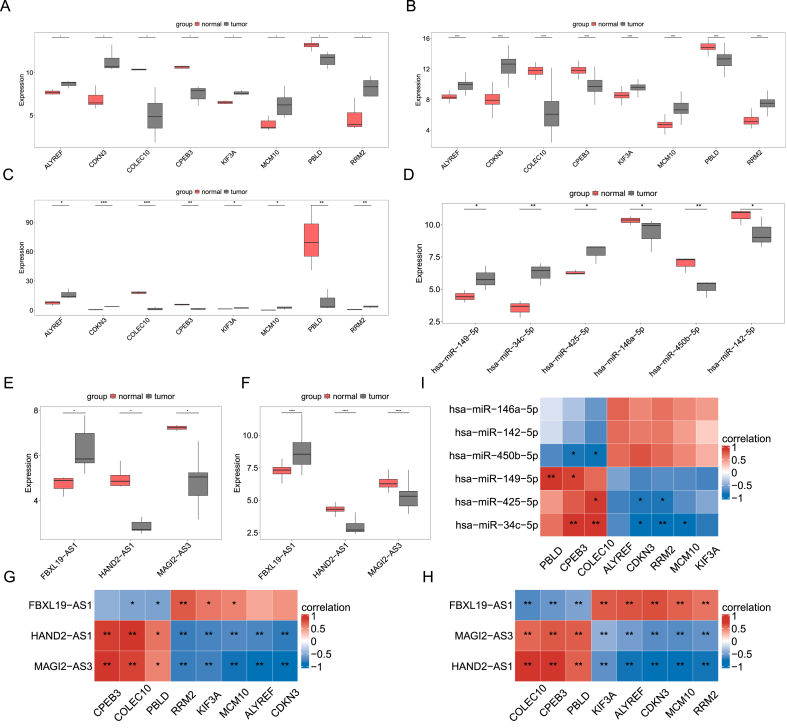
**Expression of mRNAs, miRNAs, and lncRNAs in HCC samples.** The expression of ALYREF, CDKN3, COLEC10, CPEB3, KIF3A, MCM10, PBLD and RRM2 in HCC and normal samples in the GSE101728 (A), GSE138178 (B), and GSE146719 (C) datasets. (D) The expression of six miRNAs in HCC and normal samples in the GSE146719 dataset. The expression of FBXL19-AS1, HAND2-AS1, and MAGI2-AS3 in HCC and normal samples in the GSE101728 (E) and GSE138178 (F) datasets. (G–H) The correlation between lncRNAs and mRNAs. (I) The correlation between miRNAs and mRNAs. ∗*p* < 0.05; ∗∗*p* < 0.01; ∗∗∗*p* < 0.001; ∗∗∗∗*p* < 0.0001.

Next, we analyzed the expression of eight mRNAs, six miRNAs, and three lncRNAs in the HCC samples. In the GSE101728, GSE138178 and GSE146719 datasets, the expression of ALYREF, CDKN3, KIF3A, MCM10, and RRM2 was significantly increased in HCC samples compared to normal samples, whereas the expression of COLEC10, CPEB3, and PBLD was markedly decreased (*p* < 0.05; Fig. 5A–5C). In the GSE146179 dataset, six miRNAs also showed expression differences between HCC and normal groups. The expression of hsa-miR-149-5p, hsa-miR-34c-5p, and hsa-miR-425-5p was increased in HCC samples compared to normal samples. In contrast, the expression of hsa-miR-146a-5p, hsa-miR-450b-5p and hsa-miR-142-5p was markedly decreased (Fig. 5D). Furthermore, in the GSE101728 and GSE138178 datasets, FBXL19-AS1 expression was significantly elevated, while HAND2-AS1 and MAGI2-AS3 expression were significantly reduced in HCC samples compared to normal samples (*p* < 0.05; Fig. 5E,F).

### 3.8 Correlation analysis of lncRNAs-mRNAs and miRNAs-mRNAs

In the GSE101728 dataset, we observed that the expression of FBXL19-AS1 exhibited a negative correlation with COLEC10 and PBLD while showing a positive correlation with RRM2, KIF3A, and MCM10 (Fig. 5G). HAND2-AS1 and MAGI2-AS3 expression demonstrated positive associations with CPEB3, COLEC10, and PBLD, and negative correlations with RRM2, KIF3A, MCM10, ALYREF, and CDKN3 expression (Fig. 5G). In the GSE138178 dataset, FBXL19-AS1 expression was negatively correlated with CPEB3, COLEC10, and PBLD expression and positively correlated with RRM2, KIF3A, MCM10, ALYREF, and CDKN3 expression. Conversely, expression of HAND2-AS1 and MAGI2-AS3 was positively associated with CPEB3, COLEC10, and PBLD expression, while negatively correlated with RRM2, KIF3A, MCM10, ALYREF, and CDKN3 expression (Fig. 5H). Furthermore, in the GSE146719 dataset, hsa-miR-450b-5p expression displayed a significant negative correlation with CPEB3 and COLEC10 expression, whereas hsa-miR-149-5p expression showed a significant positive correlation with PBLD and CPEB3 expression (Fig. 5I). Hsa-miR-425-5p expression was positively correlated with COLEC10 expression and negatively correlated with CDKN3 and RRM2 expression (Fig. 5I). In addition, hsa-miR-34c-5p expression exhibited a strong positive correlation with CPEB3 and COLEC10 expression, while being negatively correlated with CDKN3, RRM2, and MCM10 expression (Fig. 5I). These results suggest that the MAGI2-AS3/hsa-miR-450b-5p/COLEC10 axis might play a crucial role in the progression of HCC.

## 4 Discussion

Despite significant advancements in treatment methods, the prognosis for HCC remains bleak.^3^ This is primarily because HCC is often diagnosed at intermediate to advanced stages, with only a limited number of patients qualifying for curative treatment.^20^^,^^21^ Emerging evidence has implicated lncRNAs in various biological functions, suggesting roles in regulating multistep biological processes across multiple diseases, particularly cancer.^22^^,^^23^ The interaction between lncRNAs and mRNA may be mediated by miRNAs, where lncRNAs act as miRNA sponges, inhibiting miRNA activity and consequently leading to the derepression of mRNA expression.^24^ In the present study, we established a ceRNA network affecting HCC development. We identified DEmRNAs, DEmiRNAs, and DElncRNAs in HCC and found that high MCM10, CDKN3, RRM2, KIF3A, and ALYREF levels predict poor prognosis, while high CPEB2, COLEC10, and PBLD levels indicate better outcomes. GO and KEGG analyses and PPI networks were used to explore gene function. This ceRNA network reveals an HCC molecular pathway and may aid in discovering new biomarkers and targets for treatment, benefiting diagnosis, prognosis, and treatment development.

A total of 106 prognosis-related mRNAs were identified through univariate Cox regression analysis. GO and KEGG analyses revealed that these 106 genes were primarily enriched serine hydrolase activity, serine-type peptidase activity, glycolysis/gluconeogenesis, and fatty acid degradation. Serine metabolism is activated in various cancer types. In HCC tissues, serine levels are elevated, while phosphoglycerate dehydrogenase (PHGDH) expression is down-regulated.^25^ This down-regulation correlates with a notable increase in the catalytic activity of PHGDH in HCC tissues compared to normal tissues, which subsequently stimulates serine synthesis.^25^ The balance between glycolysis and gluconeogenesis is crucial for the metabolic regulation of HCC. Research has indicated that the enhancement of glycolysis is often accompanied by the inhibition of gluconeogenesis and vice versa.^26^^,^^27^ Gluconeogenesis, a metabolic process essential for hepatocytes, is downregulated in HCC.^28^ HCC cells adapt to their microenvironment by modulating gluconeogenesis activity, allowing tumor cells to thrive under varying metabolic conditions. For instance, by regulating PEPCK1, a key enzyme in gluconeogenesis, through Nur77, HCC cells can transition from glycolysis to gluconeogenesis under specific circumstances, thereby enhancing their survival.^28^ Fatty acid metabolism is also critical in HCC. Research has demonstrated that in high-fat diet-induced HCC, the FAO pathway is frequently down-regulated to protect HCC cells from lipotoxicity.^29^ Concurrently, de novo lipogenesis (DNL) progressively increases from normal liver tissue to liver tumors. This increase in DNL is associated with advanced HCC and is indicative of a poorer prognosis for patients.^30^ Therefore, an in-depth study of metabolic processes, including amino acid metabolism, sugar metabolism, and fatty acid metabolism, may reveal new targets for the diagnosis, prognosis assessment, and development of treatment strategies for HCC.

ALYREF,^31^ CDKN3,^32^ KIF3A,^33^ MCM10,^34^ RRM2,^35^ COLEC10,^36^ CPEB3,^37^ and PBLD^38^^,^^39^ have been reported to play critical roles in the progression and prognosis of HCC. Our findings were consistent with these reports: high expression levels of MCM10, CDKN3, RRM2, KIF3A, and ALYREF were correlated with poor prognosis, whereas elevated expression of CPEB2, COLEC10, and PBLD was associated with better prognosis in HCC patients. COLEC10, a C-type lectin mainly expressed in the liver, is involved in HCC development. COLEC10 is an independent prognostic factor that influences the overall survival of patients with HCC.^36^ The overexpression of COLEC10 reduces the growth and migratory capabilities of HCC cells in vitro and in vivo while also modulating GRP78-mediated endoplasmic reticulum stress, thereby inhibiting HCC progression through signal transduction pathways.^40^ Furthermore, COLEC10 levels exhibit a negative correlation with HCC stem cell function. COLEC10 suppresses HCC stemness by downregulating the Wnt/β-catenin pathway, which represents a promising target for liver cancer stem cell therapy.^41^ Additionally, COLEC10 has been identified as a target of miR-452-5p and miR-224-5p in HCC. The overexpression of COLEC10 can mitigate the stimulatory effects of miR-452-5p on HCC cells,^42^ while miR-224-5p negatively regulates COLEC10 expression. Notably, silencing COLEC10 can counteract the inhibitory effect of silencing miR-224-5p on HCC progression.^43^ Our study found that COLEC10 expression negatively correlated with has-miR-450b-5p in HCC. hsa-miR-450b-5p is highly expressed in hepatocellular carcinoma.^44^ Furthermore, hsa-miR-450b-5p may inhibit the progression of hepatocellular carcinoma cells by targeting and inhibiting KIF26B or RPLP0.^45^^,^^46^ Therefore, we hypothesize that hsa-miR-450b-5p may regulate HCC progression by regulating COLEC10.

According to the ceRNA hypothesis, miRNAs negatively regulate mRNA expression, while lncRNAs function as miRNA sponges, alleviating the inhibitory effects of miRNAs on target genes.^47^^,^^48^ In our study, we observed that MAGI2-AS3 was down-regulated in HCC, and we identified a positive correlation between MAGI2-AS3 and COLEC10 in HCC. Yin et al. reported that MAGI2-AS3 was down-regulated in HCC tissues and closely associated with various clinical characteristics and poor overall survival.^49^ Further analysis demonstrated that the overexpression of MAGI2-AS3 can inhibit the proliferation and migration of liver cancer cells by targeting the miR-374b-5p/SMG1 signaling pathway.^49^ In HCC, MAGI2-AS3 positively regulates FOXO1 expression by inducing miR-374a/b-5p.^50^ Additionally, MAGI2-AS3 is down-regulated in cases of distant recurrence following surgical resection of HCC, influencing the migration and invasion of HCC cells by regulating ROCK2.^51^ Accordingly, we hypothesize that the aberrantly expressed ceRNAs MAGI2-AS3/hsa-miR-450b-5p/COLEC10 may play a crucial role in HCC; however, elucidation of the specific regulatory mechanisms warrant further investigation through in vivo and in vitro studies.

Our study has several limitations that warrant consideration. Although univariate Cox regression analysis has provided valuable insights into the associations between individual variables and the outcome of interest, it does not account for potential confounding effects from multiple variables simultaneously. Furthermore, we acknowledge the absence of validation using clinical patient data, an area we are actively pursuing. We are currently collecting and integrating real-world clinical data from patients enrolled in our department, which will be compiled into a comprehensive table that details the clinical and pathological features of our patient cohort.

## 5 Conclusion

This study utilized integrated bioinformatics to uncover ceRNA networks and potential biomarkers in HCC. Through comprehensive bioinformatic analysis, we identified DEmRNAs, DEmiRNAs, and DElncRNAs between HCC and normal tissue samples and further identified mRNAs associated with the prognosis of HCC patients through univariate Cox regression analysis. A ceRNA network involving three lncRNAs, six miRNAs, and eight mRNAs was established, highlighting MCM10, CDKN3, RRM2, KIF3A, and ALYREF as poor prognosis markers, and CPEB2, COLEC10, and PBLD as more favorable prognosis markers. Furthermore, correlation analysis revealed that the MAGI2-AS3/hsa-miR-450b-5p/COLEC10 axis might play a crucial role in the progression of HCC. These findings provide valuable insights into the molecular mechanisms underlying HCC and may contribute to identifying novel biomarkers and therapeutic targets for this aggressive malignancy.

## CRediT authorship contribution statement

**Lan-Qing Yao:** Writing – original draft, Methodology, Funding acquisition, Formal analysis, Data curation, Conceptualization. **Yong-Kang Diao:** Resources, Funding acquisition, Formal analysis, Data curation, Conceptualization. **Jin-Bo Gong:** Formal analysis, Data curation, Conceptualization. **Li-Hui Gu:** Formal analysis, Data curation, Conceptualization. **Jia-Hao Xu:** Data curation, Conceptualization. **Ming-Da Wang:** Writing – original draft, Visualization, Resources, Project administration, Methodology, Investigation, Funding acquisition, Formal analysis, Data curation, Conceptualization. **Chao Li:** Writing – review & editing, Writing – original draft, Visualization, Validation, Resources, Project administration, Methodology, Investigation, Funding acquisition, Formal analysis, Data curation, Conceptualization.

## Informed consent

Not applicable.

## Data Availability Statement

The data that support the conclusions of this study can be made available by the corresponding author upon reasonable request.

## Ethics statement

Not applicable.

## Funding

This study was funded by Special clinical project of 10.13039/100017950Shanghai Municipal Health Commission (No. 20224Y0299 and No. 20244Y0233) and National Natural Science Foundation Incubation Project (No. 2022MS039 and 2022GZR005), the National Natural Science Foundation of China (82372813 for Wang MD), the Natural Science Foundation of Shanghai (No. 22ZR1477900 for Wang MD) and Shanghai Science and Technology Committee Rising-Star Program (No. 22QA1411600 for Wang MD; 24YF2758600 for Diao YK).

## Declaration of competing interest

The authors declare no conflict of interest, and the funding sources had no role in the design and conduct of the study; collection, management, analysis, and interpretation of the data; preparation, review, or approval of the manuscript; and decision to submit the manuscript for publication.

## Declaration of Generative AI and AI-assisted technologies in the writing process

Not applicable.

## References

[1] Bray F., Laversanne M., Sung H. (2024). Global cancer statistics 2022: GLOBOCAN estimates of incidence and mortality worldwide for 36 cancers in 185 countries. CA Cancer J Clin.

[2] Chayanupatkul M., Omino R., Mittal S. (2017). Hepatocellular carcinoma in the absence of cirrhosis in patients with chronic hepatitis B virus infection. J Hepatol.

[3] Yang J.D., Hainaut P., Gores G.J. (2019). A global view of hepatocellular carcinoma: trends, risk, prevention and management. Nat Rev Gastroenterol Hepatol.

[4] Jonas S., Bechstein W.O., Steinmüller T. (2001). Vascular invasion and histopathologic grading determine outcome after liver transplantation for hepatocellular carcinoma in cirrhosis. Hepatology.

[5] Hasegawa K., Kokudo N., Makuuchi M. (2013). Comparison of resection and ablation for hepatocellular carcinoma: a cohort study based on a Japanese nationwide survey. J Hepatol.

[6] Liu X.L., Wang X.H., Yu L.H. (2022). A novel prognostic score based on artificial intelligence in hepatocellular carcinoma: a long-term follow-up analysis. Front Oncol.

[7] Roayaie S., Jibara G., Tabrizian P. (2015). The role of hepatic resection in the treatment of hepatocellular cancer. Hepatology.

[8] Qian K., Chen M.J., Zhang F. (2021). Image-guided radiofrequency hyperthermia (RFH)-enhanced direct chemotherapy of hepatic tumors: the underlying biomolecular mechanisms. Front Oncol.

[9] Tagliamonte M., Petrizzo A., Mauriello A. (2018). Potentiating cancer vaccine efficacy in liver cancer. OncoImmunology.

[10] Reveron-Thornton R.F., Teng M.L.P., Lee E.Y. (2022). Global and regional long-term survival following resection for HCC in the recent decade: a meta-analysis of 110 studies. Hepatol Commun.

[11] Xue S.T., Zheng B., Cao S.Q. (2022). Long non-coding RNA LINC00680 functions as a *CeRNA* to promote esophageal squamous cell carcinoma progression through the miR-423-5p/PAK6 axis. Mol Cancer.

[12] Braga E.A., Fridman M.V., Moscovtsev A.A. (2020). LncRNAs in ovarian cancer progression, metastasis, and main pathways: *CeRNA* and alternative mechanisms. Int J Mol Sci.

[13] Akhbari M.H., Zafari Z., Sheykhhasan M. (2022). Competing endogenous RNAs (ceRNAs) in colorectal cancer: a review. Expet Rev Mol Med.

[14] Wu B., Xue X.K., Lin S.M. (2022). LncRNA LINC00115 facilitates lung cancer progression through miR-607/ITGB1 pathway. Environ Toxicol.

[15] Matboli M., Shafei A.E., Ali M.A. (2021). Role of extracellular LncRNA-SNHG14/miRNA-3940-5p/NAP12 mRNA in colorectal cancer. Arch Physiol Biochem.

[16] Wang J., Ding Y.R., Wu Y.Y. (2020). Identification of the complex regulatory relationships related to gastric cancer from lncRNA-miRNA-mRNA network. J Cell Biochem.

[17] Shou J.X., Gao H.D., Cheng S. (2021). LncRNA HOXA-AS2 promotes glioblastoma carcinogenesis by targeting miR-885-5p/RBBP4 axis. Cancer Cell Int.

[18] Wu H.L., Liu B., Chen Z.S. (2020). MSC-induced lncRNA HCP5 drove fatty acid oxidation through miR-3619-5p/AMPK/PGC1α/CEBPB axis to promote stemness and chemo-resistance of gastric cancer. Cell Death Dis.

[19] Xu Y., Luan G.C., Li Z.C. (2023). Tumour-derived exosomal lncRNA SNHG16 induces telocytes to promote metastasis of hepatocellular carcinoma *via* the miR-942-3p/MMP9 axis. Cell Oncol.

[20] Kumada T., Toyoda H., Tada T. (2014). High-sensitivity Lens culinaris agglutinin-reactive alpha-fetoprotein assay predicts early detection of hepatocellular carcinoma. J Gastroenterol.

[21] Cucchetti A., Elshaarawy O., Han G.H. (2023). ‘Potentially curative therapies' for hepatocellular carcinoma: how many patients can actually be cured?. Br J Cancer.

[22] Xue X., Yang Y.A., Zhang A. (2016). LncRNA HOTAIR enhances ER signaling and confers tamoxifen resistance in breast cancer. Oncogene.

[23] Yang X.Z., Cheng T.T., He Q.J. (2018). LINC01133 as *CeRNA* inhibits gastric cancer progression by sponging miR-106a-3p to regulate APC expression and the Wnt/β-catenin pathway. Mol Cancer.

[24] Tam C., Wong J.H., Tsui S.K.W. (2019). LncRNAs with miRNAs in regulation of gastric, liver, and colorectal cancers: updates in recent years. Appl Microbiol Biotechnol.

[25] Wang K., Luo L., Fu S.Y. (2023). PHGDH arginine methylation by PRMT1 promotes serine synthesis and represents a therapeutic vulnerability in hepatocellular carcinoma. Nat Commun.

[26] Tenen D.G., Chai L., Tan J.L. (2020). Metabolic alterations and vulnerabilities in hepatocellular carcinoma. Gastroenterol Rep.

[27] Du D.Y., Liu C., Qin M.Y. (2022). Metabolic dysregulation and emerging therapeutical targets for hepatocellular carcinoma. Acta Pharm Sin B.

[28] Bian X.L., Chen H.Z., Yang P.B. (2017). Nur77 suppresses hepatocellular carcinoma *via* switching glucose metabolism toward gluconeogenesis through attenuating phosphoenolpyruvate carboxykinase sumoylation. Nat Commun.

[29] Lin M.H., Lv D., Zheng Y.L. (2018). Downregulation of CPT2 promotes tumorigenesis and chemoresistance to cisplatin in hepatocellular carcinoma. OncoTargets Ther.

[30] Lin J., Rao D.N., Zhang M. (2024). Metabolic reprogramming in the tumor microenvironment of liver cancer. J Hematol Oncol.

[31] Wang Z.Z., Meng T., Yang M.Y. (2022). ALYREF associated with immune infiltration is a prognostic biomarker in hepatocellular carcinoma. Transl Oncol.

[32] Dai W., Miao H.L., Fang S. (2016). CDKN_3_ expression is negatively associated with pathological tumor stage and CDKN_3_ inhibition promotes cell survival in hepatocellular carcinoma. Mol Med Rep.

[33] Bannangkoon K., Hongsakul K., Tubtawee T. (2023). Validation of the ALBI-TAE model and comparison of seven scoring systems for predicting survival outcome in patients with intermediate-stage hepatocellular carcinoma undergoing chemoembolization. Cancer Imaging.

[34] Wan W., Shen Y., Li Q.X. (2020). MCM10 acts as a potential prognostic biomarker and promotes cell proliferation in hepatocellular carcinoma: integrated bioinformatics analysis and experimental validation. Cancer Manag Res.

[35] Qin Z.Q., Xie B., Qian J.Y. (2023). Over-expression of RRM2 predicts adverse prognosis correlated with immune infiltrates: a potential biomarker for hepatocellular carcinoma. Front Oncol.

[36] Zhang B.Z., Wu H.B. (2018). Decreased expression of *COLEC10* predicts poor overall survival in patients with hepatocellular carcinoma. Cancer Manag Res.

[37] Zhang H., Zou C.D., Qiu Z.N. (2020). CPEB3-mediated MTDH mRNA translational suppression restrains hepatocellular carcinoma progression. Cell Death Dis.

[38] Li A.M., Yan Q., Zhao X.M. (2016). Decreased expression of PBLD correlates with poor prognosis and functions as a tumor suppressor in human hepatocellular carcinoma. Oncotarget.

[39] Han L., Lin X., Yan Q. (2022). PBLD inhibits angiogenesis *via* impeding VEGF/VEGFR2-mediated microenvironmental cross-talk between HCC cells and endothelial cells. Oncogene.

[40] Cai M.N., Chen D.M., Xiao L.X. (2023). COLEC10 induces endoplasmic reticulum stress by occupying GRP78 and inhibits hepatocellular carcinoma. Lab Invest.

[41] Cai M.N., Chen D.M., Chen X.R. (2024). COLEC10 inhibits the stemness of hepatocellular carcinoma by suppressing the activity of β-catenin signaling. Cell Oncol.

[42] Zheng J.X., Cheng D.M., Wu D.Y. (2021). miR-452-5p mediates the proliferation, migration and invasion of hepatocellular carcinoma cells *via* targeting COLEC10. Per Med.

[43] Chen C., Zhao J.J., Qiu B. (2024). HCC control by lycorine-based restraining of the miR-224-5p/COLEC10 axis. Pak J Pharm Sci.

[44] Shi Y., Men J.R., Sun H.L. (2022). The identification and analysis of microRNAs combined biomarkers for hepatocellular carcinoma diagnosis. Med Chem.

[45] Li H., Shen S., Chen X.L. (2019). miR-450b-5p loss mediated KIF26B activation promoted hepatocellular carcinoma progression by activating PI3K/AKT pathway. Cancer Cell Int.

[46] Meng Y.Q., Huang X.B., Zhang G.X. (2024). microRNA-450b-5p modulated RPLP0 promotes hepatocellular carcinoma progression *via* activating JAK/STAT3 pathway. Transl Oncol.

[47] Tay Y., Rinn J., Pandolfi P.P. (2014). The multilayered complexity of *CeRNA* crosstalk and competition. Nature.

[48] Ma B.C., Wang S.H., Wu W.Z. (2023). Mechanisms of circRNA/lncRNA-miRNA interactions and applications in disease and drug research. Biomed Pharmacother.

[49] Yin Z., Ma T.T., Yan J.H. (2019). LncRNA MAGI2-AS3 inhibits hepatocellular carcinoma cell proliferation and migration by targeting the miR-374b-5p/SMG1 signaling pathway. J Cell Physiol.

[50] Wang C., Su K.K., Lin H.C. (2022). Identification and verification of a novel MAGI2-AS3/miRNA-374-5p/FOXO1 network associated with HBV-related HCC. Cells.

[51] Fang G., Wang J.J., Sun X.W. (2020). LncRNA MAGI2-AS3 is downregulated in the distant recurrence of hepatocellular carcinoma after surgical resection and affects migration and invasion via ROCK2. Ann Hepatol.

